# Effect of elevated atmospheric CO_2_ concentration on growth and leaf litter decomposition of *Quercus acutissima* and *Fraxinus rhynchophylla*

**DOI:** 10.1371/journal.pone.0171197

**Published:** 2017-02-09

**Authors:** Sangsub Cha, Hee-Myung Chae, Sang-Hoon Lee, Jae-Kuk Shim

**Affiliations:** Department of Life Science, Chung-Ang University, Seoul, Korea; Loyola University Chicago, UNITED STATES

## Abstract

The atmospheric carbon dioxide (CO_2_) level is expected to increase substantially, which may change the global climate and carbon dynamics in ecosystems. We examined the effects of an elevated atmospheric CO_2_ level on the growth of *Quercus acutissima* and *Fraxinus rhynchophylla* seedlings. We investigated changes in the chemical composition of leaf litter, as well as litter decomposition. *Q*. *acutissima* and *F*. *rhynchophylla* did not show differences in dry weight between ambient CO_2_ and enriched CO_2_ treatments, but they exhibited different patterns of carbon allocation, namely, lower shoot/root ratio (S/R) and decreased specific leaf area (SLA) under CO_2_-enriched conditions. The elevated CO_2_ concentration significantly reduced the nitrogen concentration in leaf litter while increasing lignin concentrations and carbon/nitrogen (C/N) and lignin/N ratios. The microbial biomass associated with decomposing *Q*. *acutissima* leaf litter was suppressed in CO_2_ enrichment chambers, while that of *F*. *rhynchophylla* was not. The leaf litter of *Q*. *acutissima* from the CO_2_-enriched chambers, in contrast with *F*. *rhynchophylla*, contained much lower nutrient concentrations than that of the litter in the ambient air chambers. Consequently, poorer litter quality suppressed decomposition.

## Introduction

In terrestrial ecosystems, carbon dioxide (CO_2_) is absorbed or emitted through primary production and respiration processes [1–3]. The amount of carbon (C) stored in terrestrial ecosystems is approximately 2,060 Gt, which is roughly three times that in the atmosphere (735 Gt) [1, 4, 5]. In particular, temperate forests cover only 8% of the land area globally, but they account for approximately 40% of the total terrestrial C storage. These forests are important for sequestering C from atmospheric CO_2_ [6–8].

Atmospheric concentrations of CO_2_ have increased substantially since the Industrial Revolution as a result of human activity [9]. The CO_2_ concentration in the atmosphere reached 397 ppm in 2014 [10]. The rising CO_2_ concentration may induce global warming [9, 11–13], and it has had direct impacts on the structure and function of ecosystems, including effects on tree physiology and growth [4, 14–16]. For example, plant biomass increases under elevated CO_2_ conditions [16, 17], as does forest productivity [1, 18], and there have been changes in the C dynamics of various ecosystems under these conditions [19, 20]. An increasing CO_2_ concentration has increased the total amount of C cycling and distorted the global C balance in ecosystems [1, 2, 5–9]. Elevated CO_2_ concentrations stimulate photosynthesis and increase net primary production, which increases the amount of C stored in trees and soil [1, 3, 14–21], notably in mid-latitude ecosystems [18].

Many studies that have focused on aboveground tree responses have shown that CO_2_ enrichment can lead to differences in forest ecosystem structure and function through direct effects on tree physiology and growth [4, 14–16], changes in C cycling [19, 20], and possible shifts in climate [9, 11–13]. Moreover, elevated CO_2_ levels can affect litter quality by altering the nutrient concentrations of plant tissue. When grown in a CO_2_ fumigation system, plants, such as *Betula pubescens*, *Fraxinus excelsior*, and *Acer platanoides*, have lower nitrogen (N) concentrations in their tissues [22–24], which results in an increase in structural and non-structural C in leaves [24–28]. These changes in plant tissues affect decomposition and mineralization processes [29, 30] by changing litter quality parameters such as the C/N and lignin to N (lignin/N) ratios [31, 32]. Consistent negative correlations with litter decomposition rates have been reported for C/N [31] and lignin/N ratios [32]. Lignin is one of the most abundant biopolymers, and it is resistant to decomposition [33, 34]. Therefore, elevated CO_2_ concentrations have the potential to modify litter decomposition by changing the quality and quantity of litter, which further affects the rates of biogeochemical processes in forest environments.

Recently, several long-term experiments using free-air CO_2_ enrichment (FACE) systems have shown the effects of a rising atmospheric CO_2_ concentration on terrestrial ecosystems. FACE experiments have provided novel insights into the ecological mechanisms that control the cycling and storage of C in ecosystems. However, in South Korea, FACE systems do not exist, and there is less concern about the effects of elevated CO_2_ concentrations on forest ecosystem function, although many studies have shown that plant growth and litter quality in forest ecosystems will change with elevated CO_2_ concentration. The aim of the present experiment was to determine the impact of elevated CO_2_ concentrations on plant growth and changes in the litter quality of two tree species, *Quercus acutissima* and *Fraxinus rhynchophylla*, which are the dominant and most widespread species in lowland temperate forests in South Korea; the acorns from *Q*. *acutissima* are traditionally used for food, while the wood from both species is used for charcoal and architectural materials [35]. Moreover, we examined the changes in the litter decomposition rate and microbial activities that were caused by CO_2_-mediated changes in litter qualities. To achieve these aims, we surveyed: (1) changes in growth, litter quality, and the chemical composition of leaf litter in elevated CO_2_ experimental chamber; (2) and changes in the decomposition rate of litter that was collected from the elevated CO_2_ chamber. We anticipate that the results from these experiments will provide a basis for studying the effects of elevated CO_2_ concentration on temperate deciduous forests in South Korea.

## Materials and methods

### Raising plants and litter collection

The acorns of *Q*. *acutissima* were germinated in a 25°C incubator, and we acquired 1-year-old *F*. *rhynchophylla* seedlings from the Korea National Arboretum of the Korea Forest Service. We transplanted the seedlings of both species in rectangular pots and maintained them for one growing season in an ambient CO_2_ (380 ppm) chamber and an enriched atmospheric CO_2_ (700 ppm) chamber; 700 ppm is the predicted CO_2_ concentration in the next century, as determined by the Intergovernmental Panel on Climate Change in 2013. The pots (0.6 m long × 0.4 m wide × 0.3 m high) were filled with artificial soil (TKS2 Instant Plus, Floragard, Oldenburg, Germany) and a vermiculite (Verminuri, GFC, Hongseong, South Korea) mixture (2:1, v/v), and each pot was fertilized once with 330 mg L^−1^ N as (NH4)_2_SO_4_, 220 mg L^−1^ P as NaH_2_PO_4_, and 400 mg L^−1^ K as KCl in the form of granular and incorporated into potting soil before transplanting the seedlings. We used four pots × two experimental tree species × two CO_2_ treatments, which resulted in a total of 16 pots. Ten *Q*. *acutissima* seedlings and six *F*. *rhynchophylla* seedlings were transplanted in each pot, which resulted in a total of 40 and 24 seedlings, respectively, for each treatment.

The seedlings in the pots were maintained in two closed-top chamber fumigation systems (2.4 m long × 1.2 m wide × 1.5 m high), one for the CO_2_ treatment and one for the ambient treatment. These chambers were constructed using polycarbonate (PC) sheet (Polygal Plastic Industries Ltd., Ramat Hashofet, Israel), in a greenhouse. The elevated CO_2_ treatment chamber was maintained at 700–750 ppm CO_2_ by constantly injecting a mixture of ambient air and a high concentration of CO_2_. The inlet ventilation air fan system forced the air into the chambers at a rate of two air changes per min. Both the chambers were ventilated in the same way, the only difference being the addition of CO_2_ for the CO_2_ treatment chamber. The system provided a stable CO_2_ concentration, and the temperature and humidity were close to those outside of the chambers. The CO_2_ concentration was monitored using an infrared CO_2_ analyzer (LI-840, LI-COR, Lincoln, NE, USA). The daytime air temperature in the chamber was at the most 2.4°C higher than the temperature outside (during the summer), and it was similar to that outside the chambers at night. The relative illumination of the chamber was 0.60–0.65 of that outside of the greenhouse. The relative illumination was calculated by simultaneously measuring the illumination inside the chamber and outside the greenhouse using a digital lux meter (DX-100, INS enterprise, Taipei, Taiwan) under full sunlight condition. The experiment used isolative segregation design with pseudoreplication in the two growth chambers [36]. This design is likely to represent the risks of simple segregation in an exaggerated form, and therefore, the potential for spurious effect of the treatment is much greater. Hence, we changed the position of the pots once a month between the two growth chambers to minimize the potential source of confusion caused by the experimental design [36, 37].

Plants were cultivated for 251 d from April 3, 2007, to December 10, 2007. Senescent leaves from the ambient and CO_2_-elevated chambers were separately collected as they fell. The collected leaves were dried at 60°C for 48 h and then stored in desiccators. The thickness of the fallen leaves from each growth pot was measured using an outside micrometer (M110-25, Mitutoyo, Kawasaki, Japan). The leaf area was measured by a scanner (Perfection 1670, Seiko Epson, Nagano, Japan) and AutoCAD (2007, Autodesk), and the leaf weight was also measured (ED423S, Sartorius, Göttingen, Germany). The leaf weight of each individual tree was calculated using the relationship between the stem weight and leaf litter weight in the pots, because it was not possible to distinguish the litter from any particular tree, and the leaf area per individual tree was determined using the average value of the area per weight of the leaves. All analyses were conducted, using an average value per tree, with each pot as a replicate. After all of the leaves had fallen, the plants were harvested and divided into aboveground and underground portions, and then they were dried completely in an oven at 60°C. Growth analysis parameters (Table 1) were calculated as follows [38].

$$Shoot-root\;ratio\;(g\;g^{-1})=total\;shoot\;weight\;(g)\;/\;total\;root\;weight\;(g)$$

$$Leaf\;weight\;ratio\;(g\;g^{-1})=total\;leaf\;weight\;(g)\;/\;total\;plant\;weight\;(g)$$

$$Specific\;leaf\;area\;(cm^{2}\;g^{-1})=total\;leaf\;area\;(cm^{2})\;/\;total\;leaf\;weight\;(g)$$

**Table 1:** Comparison of the growth parameters of *Q*. *acutissima* and *F*. *rhynchophylla* in the ambient air (380 ppm) and elevated CO_2_ (700 ppm) chambers.

	*Q*. *acutissima*			*F*. *rhynchophylla*		
Plant growth parameters	Ambient air	Elevated CO_2_	*p*	Ambient air	Elevated CO_2_	*p*
Total dry weight (g)	14.77±2.06	15.35±3.59	0.892	29.5±4.60	28.9±1.71	0.914
S/R	0.51±0.009	0.35±0.031	0.003	1.16±0.06	0.98±0.16	0.307
LWR	0.16±0.002	0.14±0.004	0.001	0.11±0.002	0.13±0.008	0.028
Thickness (mm)	0.09±0.002	0.11±0.003	0.002	0.13±0.002	0.15±0.001	<0.001
Leaf area (cm^2^)	34.90±2.30	32.23±3.14	0.518	34.74±2.48	24.23±2.79	0.030
SLA (cm^2^ g^-1^)	235.1±8.79	176.7±5.93	0.001	241.1±5.96	181.9±2.19	<0.001

Values are means ± SE.

### Litter decomposition in microcosms

The decomposition experiment used leaf litter that was collected from the ambient air and elevated CO_2_ chambers, and it was conducted in a microcosms that were created within 1-L, colorless glass bottles with detachable lids, with a 9-mm diameter aperture attached to a rubber septum. The bottles were filled with 400 g of quartz sand from which organic matter was removed via repeated washing with distilled water. The quartz sand was controlled at 90% of its water-holding capacity to maintain constant humidity in the microcosm. Approximately 2 g of dried litter from the ambient and elevated CO_2_ chambers was enclosed in each litter bag (10 cm × 10 cm; composed of polyvinyl chloride-coated fiberglass net with a 1.2 mm^2^ mesh size), which were placed on the sand in each microcosm after immersion in a soil suspension solution (fresh soil:water = 1:20, weight/volume). The microcosms were incubated in a room at constant temperature (23°C) for 255 d. The litter bags were collected five times at approximately 50-d intervals during the incubation period (45, 90, 135, 195 and 255 days, respectively). The experimental design consisted of two species × two treatments × five collections × four replicates, giving a total 80 bags and bottles. The decaying litter was carefully removed from the litter bags, and then it was divided into two parts, one of which was dried at 60°C for 48 h to determine the remaining mass, while the other was used to measure the microbial biomass. The decomposition rate constant (*k*) was estimated following the exponential model [39] that characterizes the weight loss.

$$k=\text{-}[\ln\;(X_{t}/X_{0})]/t,$$

where *X*_0_ = weight (g) of litter at time 0; *X*_t_ = weight (g) of litter at time t (days); *k* = decomposition rate constant.

### Measurement of microbial respiration of decaying litter

CO_2_ evolution from the decaying litter was measured at 3-d intervals for the first 97 d, and then at 7–14-d intervals thereafter. The microcosms were thoroughly flushed with CO_2_-free air before the evolved CO_2_ was collected from the decaying litter. The microcosms were incubated at 23°C for 2 h with completely closed lids, and then the evolved CO_2_ was sampled by inserting a syringe through the rubber septum in the lids. The CO_2_ concentrations were determined using an infrared CO_2_ analyzer (modification of LI-840, LI-COR, Lincoln, NE, USA).

### Microbial biomass C and N in decaying litter

Fungal (eukaryotic) and bacterial (prokaryotic) biomasses in the decaying litter were determined by the substrate-induced respiration (SIR) method, as described in Beare et al. [40], as adapted from Anderson and Domsch [41]. Chopped fresh litter samples (0.2–0.5 g) were placed into four 100-mL bottles and incubated at 4°C, followed by 2.5 mL g^−1^ additions of one the following solutions: (1) cycloheximide (16.0 g L^−1^), (2) streptomycin (3.2 g L^−1^), (3) cycloheximide (16.0 g L^−1^) + streptomycin (3.2g L^−1^), or (4) pure water only. Cycloheximide and streptomycin were used as eukaryotic and prokaryotic inhibitors, respectively. Additionally, 2.5 mL g^−1^ of a glucose solution (16.0 g L^−1^) was added to the bottles. The bottles were immediately sealed and incubated at 23°C for 2 h. After incubation, evolved CO_2_ from the litter was measured using an infrared CO_2_ analyzer as described above. Pure water-treated microbial biomass carbon (C_mic_) was calculated using the formula of Beare et al. [40].

$$C_{mic}\;(\mu gC\;g^{\text{-}1}\;dry\;litter)=14.3\;SIR\;rate\;(\mu g\;CO_{2}\;\text{-}\;C\;g^{\text{-}1}\;dry\;litter\;h^{-1})-\;765.1$$

Bacterial and fungal contributions to the total biomass were calculated using the aforementioned streptomycin and cycloheximide treatments [40, 42].

$$Bacterial\;SIR\;(\%\;of\;total\;SIR)=[\{(R-R_{B})+(R_{F}-R_{BF})\}/2]/(R-R_{BF})*100$$

$$Fungal\;SIR\;(\%\;of\;total\;SIR)=[\{(R-R_{F})+(R_{B}-R_{BF})\}/2]/(R-R_{BF})*100$$

*R*_*B*_ is the streptomycin-treated SIR rate; *R*_*F*_ is the cycloheximide-treated SIR, and *R*_*BF*_ is both treated SIR rate. Fungal and bacterial biomasses were calculated according to the above equation.

Microbial biomass nitrogen (N_mic_) was determined by the modified chloroform fumigation extraction method [43]. The freshly chopped litter sample was divided into two portions, one of which was extracted with 40 mL of 0.5 M K_2_SO_4_ with shaking for 1 h, followed by filtration through Whatman filter paper (no. 42), while the other subsample was fumigated with ethanol-free chloroform for 24 h in darkness, and then the chloroform was evacuated. The samples were extracted with 0.5 M K_2_SO_4_ and filtered. A 0.75-mL aliquot of the K_2_SO_4_ extract, 1.75 mL of citric acid buffer, and 1.25 mL of freshly prepared ninhydrin reagent (composed of ninhydrin, dimethyl sulfoxide, and lithium acetate buffer) were placed in a 15-mL test tube and mixed, and then boiled for 15 min in a water bath. After cooling, 5 mL of a 1:1 water and ethanol solution was added, and the optical density was determined at 570 nm. Ninhydrin-reactive N was calculated using L-leucine standards. Microbial biomass N was calculated according to Joergensen and Brookes [44].

$$N_{mic}\;(mg\;g^{-1}\;dry\;litter)=5.0*Ninhydrin-N\;(mg\;g^{-1}\;dry\;litter)$$

### Chemical analysis of leaf litter

The chemical composition of the litter was determined on milled litter samples. To determine the nutrient concentration, the litter materials were processed by the wet digestion method using 10 mL of HNO_3_ and 3 mL of HClO_4_ [45]. The concentrations of P, K, Ca, Mg, and Na in the digested aliquots were determined by inductively coupled plasma atomic emission spectroscopy (ICPS-7510, Shimadzu Corp., Kyoto, Japan). Lignin and cellulose concentrations were determined by the acid detergent fiber / 72% H_2_SO_4_ method [46]. Approximately 0.5 g of a milled litter sample was weighed (W1) and boiled for 1 h in a 100-mL cetyltrimethylammonium bromide solution. The solution was filtered through a pre-weighed sinter (W2) and washed three times with hot distilled water. Then, it was washed with acetone, dried for 2 h at 105°C, and weighed (W3). Approximately 10 mL of 72% H_2_SO_4_ was added to the sinter, and the mixture was kept in 72% H_2_SO_4_ for 3 h. Subsequently, the acid was removed under vacuum, and the residue was washed with hot distilled water until it was acid-free (acid-detergent fiber). The sinter was dried at 105°C for 2 h and weighed (W4). Then, the sinter was heated at 550°C for 2 h, cooled, and weighed to determine the ash content of the residue (W5). Lignin (%) and cellulose (%) were calculated as follows.

%Lignin=(W4−W5)/W1∗100

%Cellulose=(W3−W4)/W1∗100

Soluble carbohydrates (CHOs) were determined by the anthrone method after hot water extraction [47]. Total C and N were determined by elemental analyzers (Flash EA 1112, Thermo Fisher Scientific, Waltham, MA, and EA1110, CE Instruments, Wigan, UK).

### Data analysis

All the data analyses were performed, using average values, with each pot considered an experimental replicate. The growth parameters and chemical concentrations of leaf litter data were analyzed for statistically significant differences between the treatments, as determined by a Student's *t*-test. Remaining mass, microbial biomass C and N, fungal and bacterial biomass data were analyzed via repeated measures ANOVAs (rmANOVAs). Each species was analyzed individually with treatment as the within subjects factor. Greenhouse-Geisser corrections were used when the assumption of sphericity was violated. Litter quality (C, N, P, K, Ca, soluble carbohydrate, cellulose, lignin, C/N, and lignin/N) and microbial biomass parameters (microbial biomass C and N, fungal and bacterial biomass, and microbial respiration) were compared using a multivariate analysis of variance (MANOVA) with the treatment of each tree species, and a 2-way MANOVA with a species × treatment interaction (represented by *Q*. *acutissima + F*. *rhynchophylla*) using SPSS for Windows, version 21 (IBM Corp., Armonk, NY, USA). Additionally, we conducted a non-metric multidimensional scaling (NMDS) analysis to examine the relationships between decaying leaf litter and initial litter quality, using metaMDS and envfit in the vegan library running the R package (ver. 2.15.3, www.r-project.org). For all analyses, differences among groups were considered significant if *p* < 0.05.

## Results

### Plant growth in the elevated CO_2_ chamber

The growth of *Q*. *acutissima* and *F*. *rhynchophylla* did not statistically differ between the ambient air and elevated CO_2_ chambers (Table 1). However, the shoot/root (S/R) ratio differed between the two species. The S/R ratio of *Q*. *acutissima* was significantly lower in elevated CO_2_ chamber. Leaf growth conspicuously differed between the conditions. The thickness of the leaf blade was significantly higher (22% for *Q*. *acutissima* and 15% for *F*. *rhynchophylla*) in the elevated CO_2_ chamber. The leaf area of *F*. *rhynchophylla* litter was significantly lower in elevated CO_2_ chamber. And the specific leaf area (SLA) of the leaf litter of *Q*. *acutissima* and *F*. *rhynchophylla* were significantly lower at the higher CO_2_ concentration (Table 1).

### Changes in the chemical composition of leaf litter

The C concentration of the leaf litter of *Q*. *acutissima* that was grown in the ambient and elevated CO_2_ chambers did not differ significantly (Table 2). However, the N concentration of the leaf litter was significantly lower in the elevated CO_2_ chamber. Conversely, in the *F*. *rhynchophylla* litter, the C concentration was higher in the elevated CO_2_ chamber, while the N concentration did not differ significantly between the two conditions. The C/N ratio of the *Q*. *acutissima* litter increased by 1.50-fold in the elevated CO_2_ chamber, and that of *F*. *rhynchophylla* litter did not significant but showed an increasing trend. The P concentration of the leaf litter differed between the two tree species. The P of the *Q*. *acutissima* litter was lower in the elevated CO_2_ chamber, while that of *F*. *rhynchophylla* was higher. However, the calcium concentration of the leaf litter was significantly lower in the elevated CO_2_ chamber for both species (Table 2). The cellulose concentration in the leaf litter of *Q*. *acutissima* was significantly lower under high CO_2_, while the soluble CHO and lignin concentrations were significantly higher in the elevated CO_2_ chamber (1.42-fold and 1.20-fold for *Q*. *acutissima*, 1.12-fold and 1.13-fold *F*. *rhynchophylla*, respectively). The lignin/N ratio of the *Q*. *acutissima* leaf litter was remarkably higher at the higher CO_2_ concentration and *F*. *rhynchophylla* leaf litter did not significant but showed an increasing tendency as C/N ratio.

**Table 2:** Chemical concentrations of leaf litter collected from the ambient air and elevated CO_2_ chambers.

	*Q*. *acutissima*			*F*. *rhynchophylla*		
Litter chemical concentration	Ambient air	Elevated CO_2_	*p*	Ambient air	Elevated CO_2_	*p*
C (%)	46.4±0.1	46.4±0.3	0.889	44.5±0.2	45.7±0.2	0.004
N (%)	0.85±0.04	0.56±0.02	0.001	0.68±0.04	0.55±0.04	0.126
P (*μ*g g^-1^)	1024.5±15.2	846.4±18.8	<0.001	539.5±19.8	649.5±9.9	0.003
K (*μ*g g^-1^)	3281.5±72.9	3042.8±58.3	0.043	10097.6±169.0	9420.5±152.3	0.025
Ca (*μ*g g^-1^)	17263±138.1	15221±128.2	<0.001	19339±280.8	16493±304.4	<0.001
C/N	55.1±2.8	82.6±3.6	0.001	66.4±3.8	84.3±4.8	0.085
Soluble carbohydrate (%)	10.7±0.7	15.2±0.6	0.002	12.6±0.2	14.1±0.2	0.002
Cellulose (%)	24.1±0.4	20.7±0.3	0.001	25.5±0.9	23.4±0.8	0.137
Lignin (%)	16.8±0.5	20.2±0.5	0.003	12.0±0.3	13.5±0.3	0.016
Lignin/N	19.9±1.0	36.1±1.8	0.001	17.8±1.5	24.6±2.8	0.068

Values are means ± SE.

### Litter decomposition

**Fig 1:**
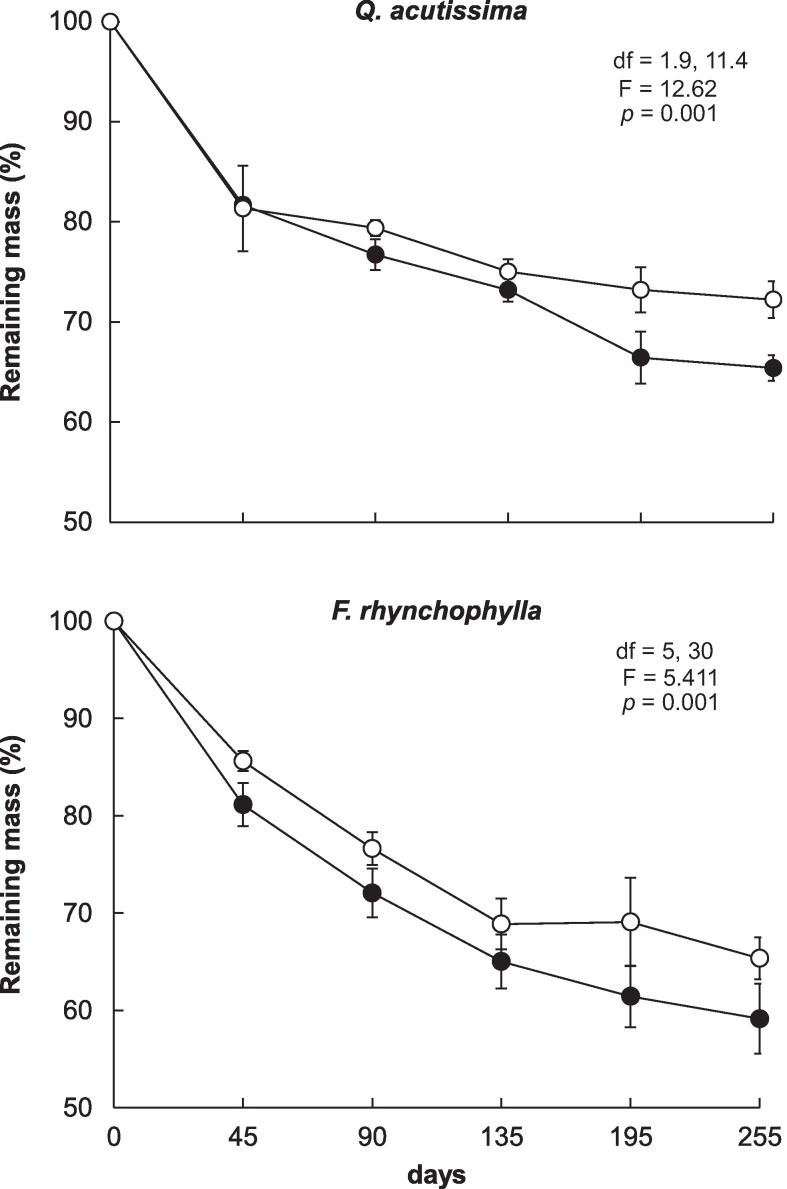
Changes in the remaining mass of the *Q*. *acutissima* (upper) and *F*. *rhynchophylla* (lower) leaf litter in the microcosms. Closed circles represent leaf litter obtained from the ambient air (380 ppm CO_2_) chamber, and open circles represent that collected from the elevated CO_2_ (700 ppm) chamber. Bars show standard deviations (n = 4).

The mass losses of the two species’ leaf litter following incubation at 23°C for 255 d in the microcosms are shown in Fig 1. The leaf litter from the elevated CO_2_ chamber exhibited a significantly lower decay rate than the litter from the ambient air chamber. The mass losses of the *Q*. *acutissima* and *F*. *rhynchophylla* litter in the ambient air chamber were 34.6% and 40.9%, respectively, and the *Q*. *acutissima* and *F*. *rhynchophylla* litter from the elevated CO_2_ chamber decomposed by 27.8% and 34.6%, respectively (Fig 1). The decomposition rate constants (*k*) of the leaf litter from the elevated CO_2_ chamber were significantly smaller than that in the ambient air chamber. The *k* values of the *Q*. *acutissima* and *F*. *rhynchophylla* litter in the ambient air chamber were 0.0014 yr^−1^ and 0.0021 yr^−1^, respectively, and those of the litter from the elevated CO_2_ chamber were 0.001 yr^−1^ and 0.0016 yr^−1^, respectively.

### Microbial biomass and activity of decaying litter

**Fig 2:**
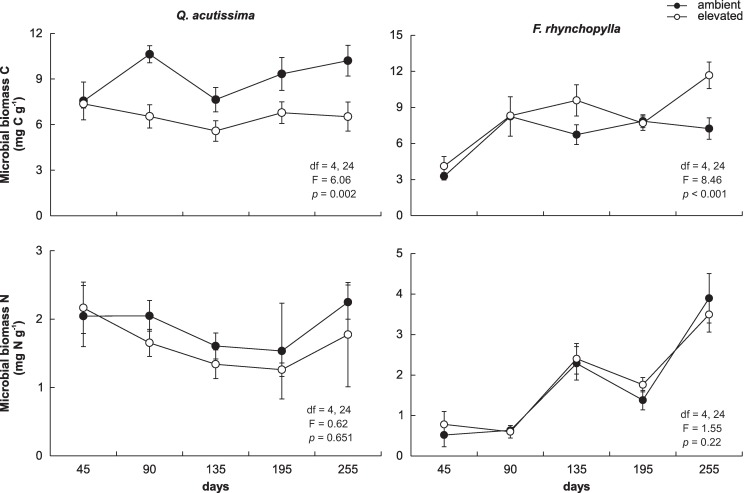
Changes in the microbial biomass C and N of the decomposing leaf litter in the microcosms. Closed circles show leaf litter obtained from the ambient air (380 ppm CO_2_) chamber, and open circles represent leaf litter obtained from the elevated CO_2_ (700 ppm) chamber. Bars show standard deviations (n = 4).

Changes in the microbial biomass C and N during the decomposition of the leaf litter of both species, which was collected from the ambient air and elevated CO_2_ chambers in the laboratory microcosm, are summarized in Fig 2. The microbial biomass C in the leaf litter of *Q*. *acutissima* was significantly greater in the litter from the ambient air chamber, compared with that from the elevated CO_2_ chamber, while the microbial biomass C in the leaf litter of *F*. *rhynchophylla* was significantly lower than elevated CO_2_ chamber. However, microbial biomass N in the leaf litter of both species did not differ significantly between the elevated CO_2_ and ambient air chambers.

There were differences in the microbial biomass change during the decomposition of the two leaf litter species. The microbial biomass C and N in the decaying *Q*. *acutissima* leaf litter were nearly stable throughout the experimental period of 255 d. However, during the decomposition of *F*. *rhynchophylla* leaf litter, the microbial biomass N increased gradually throughout the experimental period.

**Fig 3:**
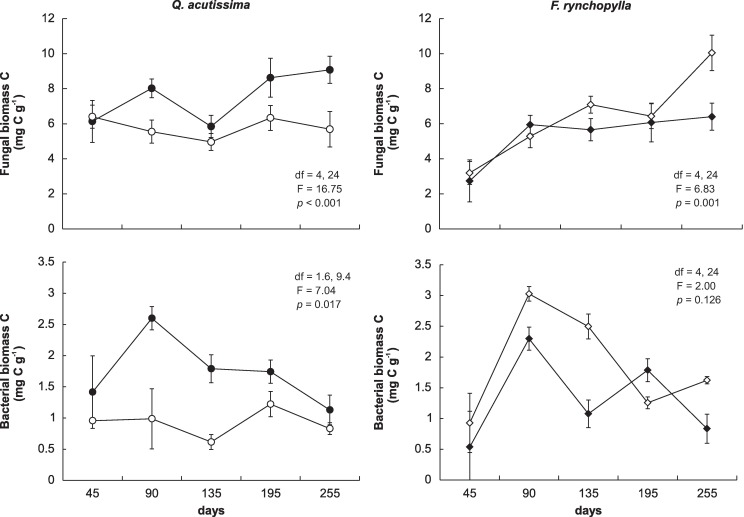
Changes in the fungal and bacterial biomasses in the decomposing leaf litter in the microcosms. Closed circles represent leaf litter obtained from the ambient air (380 ppm CO_2_) chamber, and open circles represent leaf litter obtained from the elevated CO_2_ (700 ppm) chamber. Bars show standard deviations (n = 4).

Changes in the fungal and bacterial biomasses in the decaying litter differed according to litter species and treatment (Fig 3). The bacterial biomass showed a tendency to peak 90 d into the experiment, whereas the fungal biomass peaked at the end of the experiment. In the *Q*. *acutissima* leaf litter, the fungal and bacterial biomasses were greater in the litter from the ambient air chamber, compared with that from the elevated CO_2_ chamber. However, the fungal biomass of the *F*. *rhynchophylla* leaf litter was lower in the elevated CO_2_ chamber. And bacterial biomasses of the *F*. *rhynchophylla* leaf litter occasionally varied between the ambient air and elevated CO_2_ chambers. These results suggest that there was a succession of microbial groups during the course of the experiment; the bacterial group increased during the early decay stages, while the fungal group dominated after the early stages of leaf litter decomposition for both tree species.

**Fig 4:**
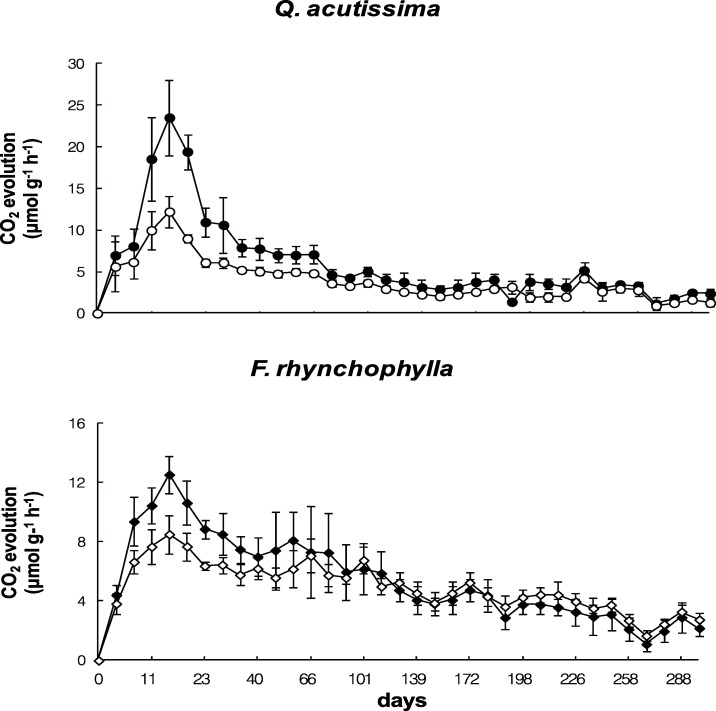
Changes in CO_2_ evolution from the decomposing leaf litter in the microcosms. Closed circles represent leaf litter obtained from the ambient air (380 ppm CO_2_) chamber, and open circles represent leaf litter obtained from the elevated CO_2_ (700 ppm) chamber. Bars show standard deviations (n = 4).

Microbial respiration in the decomposing leaf litter of the two species was active for 1 month, peaking 15 d after incubation. However, the amount of respiration was about two times greater in the *Q*. *acutissima* leaf litter than in the *F*. *rhynchophylla* litter, and it was significantly lower in the litter from the elevated CO_2_ chamber than in the ambient air chamber. Differences of microbial respiration between the two types of litter were greater at approximately 15 d of incubation, and small differences appeared gradually as litter decay progressed (Fig 4). These differences in microbial respiration between the litter from the ambient air and elevated CO_2_ chambers coincided more with the litter mass loss trends than with the parameters of microbial biomass.

### Factors affecting leaf litter decomposition

Litter quality (C, N, P, K, Ca, soluble CHO, cellulose, lignin, C/N, and lignin/N) was significantly affected by the elevated CO_2_ (Table 3). And the microbial biomass parameters of decaying *Q*. *acutissima* and *F*. *rhynchophylla* leaf litter showed also significant differences between the two treatments (Table 3).

**Table 3:** Summary of the MANOVA results for the effect of elevated CO_2_ on *Q*. *acutissima* and *F*. *rhynchophylla*.

	Litter quality			Microbial biomass		
Species	n	*F*	*p*	n	*F*	*p*
*Q*. *acutissima*	4	667.7	0.001	4	35.5	0.002
*F*. *rhynchophylla*	4	415	0.002	4	46	0.001
*Q*. *acutissima + F*. *rhynchophylla*	8	33.3	0.007	8	23.9	0.001

**Fig 5:**
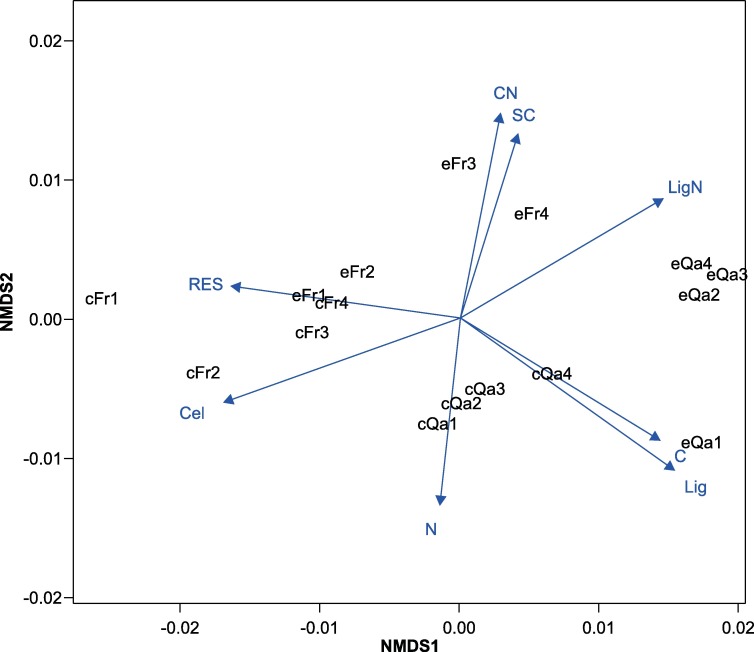
Ordination biplot of NMDS based on the leaf litter decomposition rate of each species. **Angles and lengths of the radiating arrows indicate the direction and strength of the relationships of the litter quality parameters.** Each vector's significance level was less than 0.05. Carbon (C), nitrogen (N), lignin (Lig), cellulose (Cel), soluble carbohydrate (SC), C/N (CN), lignin/N (LigN), respiration (RES), cQa (ambient CO_2_ *Q*. *acutissima* group), eQa (elevated CO_2_ *Q*. *acutissima* group), cFr (ambient CO_2_ *F*. *rhynchophylla* group), and eFr (elevated CO_2_ *F*. *rhynchophylla* group).

The results of the NMDS, which identified the relative effects of each parameter on litter decomposition, are shown in Fig 5. In the NMDS analysis, the litter from each CO_2_ treatment of *Q*. *acutissima* and *F*. *rhynchophylla* formed a distinct cluster that was related to mass loss (Fig 5). The vectors of the litter quality factors were fitted to the extent of the differences of the litter decomposition rate in ordination space. The C, N, and lignin concentrations were correlated with the ordination (*r*^*2*^ = 0.524, 0.722, and 0.902, respectively; *p* < 0.001), with arrows pointing in the direction of the *Q*. *acutissima* group. Moreover, the cellulose and soluble CHO concentrations were significantly correlated with mass loss (*r*^*2*^ = 0.802 and 0.546, respectively; *p* < 0.001), with arrows pointing toward the *F*. *rhynchophylla* group. For the C/N, lignin/N ratios, and microbial respiration, the arrows also pointed in the direction of the *F*. *rhynchophylla* group (*r*^*2*^ = 0.841, 0.902, and 0.524, respectively; *p* < 0.01). Microbial biomass C and N, fungal and bacterial biomasses did not significantly correlate with mass loss.

## Discussion

The objective of the present study was to investigate the effect of elevated CO_2_ concentration on the growth and litter quality of *Q*. *acutissima* and *F*. *rhynchophylla*, and to determine the changes in litter decomposition according to any CO_2_-mediated change in the litter quality. Our results did not show a statistically significant difference in plant growth, as evidenced by the total dry weight, between the ambient and elevated CO_2_ concentrations. The S/R ratio of the tree species was lower under elevated CO_2_. Changes in plant growth response to high CO_2_ concentrations depend on the growth conditions or growth potential of a species [16, 21, 48, 49], as well as the species’ developmental strategy, such as the creation of new sinks for extra C [50]. Several studies have suggested that growth under elevated CO_2_ concentrations leads to shifts in the root system architecture, which could enhance the nutrient uptake capacity [51–53] by increasing the biomass and density of the fine roots [3, 54–56], as well as the S/R ratio [50]. Our results are consistent with the aforementioned studies. In our experiments, the S/R ratio for *Q*. *acutissima* was clearly lower at the elevated CO_2_ concentration. It is evident that an elevated CO_2_ concentration can reduce the stem proportion and increase the root proportion, at least in young tree seedlings in some species, without changing the mass.

High atmospheric CO_2_ concentrations affect the physical structure of leaf litter [3, 57, 58]. In the present study, for both *Q*. *acutissima* and *F*. *rhynchophylla*, the leaf thickness was higher and the leaf area was lower under the elevated CO_2_ concentration, without changes in leaf mass. This is consistent with the results of many studies [59–63]. However, Pritchard et al. [64] showed variable differences according to species, highlighting that leaf thickness typically increased (81%) or was occasionally unaffected (19%) by increased CO_2_ concentrations.

Litter quality, such as the concentrations of nutrients and their composition, of the young *Q*. *acutissima* and *F*. *rhynchophylla* seedlings was also significantly affected by the elevated CO_2_ concentration (Table 3). The elevated CO_2_ treatment of the two tree seedlings altered the chemical composition of the leaf litter, e.g., it decreased the N concentration and increased the C/N ratio in *Q*. *acutissima*, and increased the lignin and soluble CHO concentrations in *Q*. *acutissima* and *F*. *rhynchophylla*. Many studies have also asserted that the N concentration of a plant tissue is reduced and the C/N ratio is increased by CO_2_ enrichment [65, 66], owing to a physiological response that increases the efficiency of N use in plants. This leads to a greater production of biomass per mole of N taken up [66, 67]. Moreover, CO_2_ enrichment generally increases the concentration of non-structural C, such as soluble CHOs [68] and secondary compounds [69, 70], through enhanced production of photosynthetic products and biomass dilution [71]. Elevated CO_2_ concentrations also increase the lignin concentration [24, 25, 28, 72–75], while decreasing the cellulose concentration in leaf litter. This, in turn, increases the lignin/N ratio significantly, which is used as a predictive index of decomposition [31, 32, 34, 72, 76]. Therefore, a decreased N concentration and increased lignin concentration and lignin/N ratio, which are induced by an increase in the atmospheric CO_2_ concentration, could reduce the litter decomposition rate [30, 34, 77, 78] by preventing an increase in microbial biomass, as well as microbial activities, by changing the litter quality [34, 79, 80].

During litter decomposition, both the chemical composition of the litter (its quality) and microbial biomass and its activity are very important factors [81, 82]. The leaf litter decomposition of the two tree species was significantly affected by changes in the litter quality that were induced by the CO_2_ treatment (Table 3). These changes in litter quality, such as the N and lignin concentrations, the C/N ratio, and the lignin/N ratio are considered to be major factors affecting litter decomposition [30–32, 34, 79]. A decrease in the N concentration and an increase in the lignin concentration change the quality of leaf litter, which affect the decomposer activities of microorganisms [83]. The microbial biomass in the litter was altered due to elevated CO_2_ treatments (Table 3), but there was not significantly affect the litter decomposition rate (Fig 5). It is presumably the result of a species-specific responses on decomposition [24]. Microbial succession [34, 77–80, 84], i.e., alteration of bacterial and fungal biomasses, differed significantly between the ambient and elevated CO_2_ treatments, but the microbial biomass and activity (respiration), which were altered by the elevated CO_2_ treatment, had weaker effects on litter decomposition than the litter quality.

In present study, although with potential error due to pseudoreplication, the litter produced under elevated CO_2_ conditions showed a tendency to decompose more slowly due to CO_2_-induced changes in the litter quality. The decreasing litter decomposition rate may expedite C deposition on the forest floor. Long-term experiments under FACE have produced findings related to the C cycle. Norby and Zak [85] reviewed the effect of CO_2_ on C cycling, and they suggested that an elevated CO_2_ concentration does not necessarily increase ecosystem C storage. However, there are many indications that C accumulates in either plant biomass or soil organic matter in certain ecosystems or under certain conditions [86–90]. By examining the effect of elevated CO_2_ conditions, compared with ambient CO_2_ conditions, Luo et al. [88] showed that the average C pools in shoots, roots, and whole plants increased by 22.4, 31.6, and 23.0%, respectively. Similarly, via a meta-analysis, they found that litter and soil C pools increased by 20.6 and 5.6% under elevated CO_2_ concentrations. Generally, forest ecosystems store a large amount of C in the form of humus. The humus can constitute as much 30 percent of the organic matter in some soil [91]. Many researchers have suggested that such an accumulation would continue for millennia in the absence of a disturbance, which would result in the buildup of considerable amounts of humus [84]. Additional reductions in decomposition rates of two species from elevated CO_2_, resulting from increased anthropogenic CO_2_ emissions, may have an impact on increase C accumulation in temperate deciduous forest in South Korea.

Based on the above results and our experimental results, the effects of increasing CO_2_ concentrations on plant growth vary by tree species (*Q*. *acutissima* and *F*. *rhynchophylla*). Nevertheless, by elevated CO_2_, more photosynthetic products were allocated to roots (which decreased the S/R ratio), and leaves became thicker and smaller at the elevated CO_2_ concentrations. Furthermore, litter quality was altered by the elevated CO_2_ concentration because of the lower N concentration or the higher lignin concentration in the leaf litter. Litter from the elevated CO_2_ treatment decayed more slowly; thus, we believe that C deposition in the soil accelerated under these conditions. In our study, we only investigated the direct effects of an elevated CO_2_ concentration. However, in addition to increasing CO_2_ concentrations, other environmental factors (climate and atmospheric environment) can affect plants [9], and these changes also impact ecosystems [9, 92–94]. Changes in factors such as temperature and precipitation have a major impact on the distribution and activity of organisms, which is a major concern of many researchers [95–101]. Recently, several studies have examined the combined effects of elevated atmospheric CO_2_ concentrations and other environmental factors, especially temperature [16, 98, 102, 103], light [104, 105], and water [106]. Nevertheless, studies of such combined effects are scarce. The combined effects of environmental factors and elevated CO_2_ concentrations on forest ecosystem are uncertain because they are very complex, especially regarding C cycling [107, 108]. Therefore, additional research is needed to understand the effects of other factors in combination with elevated CO_2_ concentrations.
